# Panobinostat Enhances Cytarabine and Daunorubicin Sensitivities in AML Cells through Suppressing the Expression of BRCA1, CHK1, and Rad51

**DOI:** 10.1371/journal.pone.0079106

**Published:** 2013-11-11

**Authors:** Chengzhi Xie, Christina Drenberg, Holly Edwards, J. Timothy Caldwell, Wei Chen, Hiroto Inaba, Xuelian Xu, Steven A. Buck, Jeffrey W. Taub, Sharyn D. Baker, Yubin Ge

**Affiliations:** 1 Department of Oncology, Wayne State University School of Medicine, Detroit, Michigan, United States of America; 2 Molecular Therapeutics Program, Barbara Ann Karmanos Cancer Institute, Wayne State University School of Medicine, Detroit, Michigan, United States of America; 3 The State Engineering Laboratory of AIDS Vaccine, College of Life Science, Jilin University, Changchun, China; 4 Pharmaceutical Sciences Department, St. Jude Children’s Research Hospital, Memphis, Tennessee, United States of America; 5 MD/PhD Program, Wayne State University School of Medicine, Detroit, Michigan, United States of America; 6 Cancer Biology Program, Wayne State University School of Medicine, Detroit, Michigan, United States of America; 7 Department of Oncology, Division of Leukemia/Lymphoma, St. Jude Children’s Research Hospital, Memphis, Tennessee, United States of America; 8 Division of Pediatric Hematology/Oncology, Children’s Hospital of Michigan, Detroit, Michigan, United States of America; 9 Department of Pediatrics, Wayne State University School of Medicine, Detroit, Michigan, United States of America; Institut national de la santé et de la recherche médicale (INSERM), France

## Abstract

Acute myeloid leukemia (AML) remains a challenging disease to treat and urgently requires new therapies to improve its treatment outcome. In this study, we investigated the molecular mechanisms underlying the cooperative antileukemic activities of panobinostat and cytarabine or daunorubicin (DNR) in AML cell lines and diagnostic blast samples *in vitro* and *in vivo*. Panobinostat suppressed expression of BRCA1, CHK1, and RAD51 in AML cells in a dose-dependent manner. Further, panobinostat significantly increased cytarabine- or DNR-induced DNA double-strand breaks and apoptosis, and abrogated S and/or G2/M cell cycle checkpoints. Analogous results were obtained by shRNA knockdown of BRCA1, CHK1, or RAD51. Cotreatment of NOD-SCID-IL2Rγ^null^ mice bearing AML xenografts with panobinostat and cytarabine significantly increased survival compared to either cytarabine or panobinostat treatment alone. Additional studies revealed that panobinostat suppressed the expression of *BRCA1, CHK1,* and *RAD51* through downregulation of E2F1 transcription factor. Our results establish a novel mechanism underlying the cooperative antileukemic activities of these drug combinations in which panobinostat suppresses expression of *BRCA1, CHK1,* and *RAD51* to enhance cytarabine and daunorubicin sensitivities in AML cells.

## Introduction

Acute myeloid leukemia (AML) remains a clinical challenge. Resistance to cytarabine (ara-C) and anthracycline [e.g., daunorubicin (DNR)]-based chemotherapy is a major cause of treatment failure in this disease [1]–[5]. Therefore, new therapies are urgently needed for this deadly disease. Histone deacetylase (HDAC) inhibitors (HDACIs) are a promising new class of anti-cancer drugs, which induce differentiation, cell cycle arrest, and apoptosis in human leukemic cells, but less so in normal cells [6]–[13]. Despite their well-characterized molecular and cellular effects [9], [14], single-agent clinical activities of HDACIs have been modest [15]–[22]. Preclinical data indicate a compelling rationale for designing drug combinations using HDACIs with other chemotherapy agents [23]. Recent clinical studies have demonstrated that vorinostat can be given safely with standard chemotherapy and the combination is active against AML [24], [25]. We previously demonstrated synergistic antileukemic interactions between valproic acid (VPA) and cytarabine in pediatric AML cells, accompanied by cooperative induction of DNA double-strand breaks (DSBs) and apoptosis [26]; however, the underlying molecular mechanisms remain largely unknown.

Our most recent studies involving the treatment of AML cell lines with structurally diverse HDACIs and shRNA knockdown of individual HDACs revealed that downregulation of both HDACs 1 and 6 is critical in enhancing cytarabine-induced apoptosis. At clinically achievable concentrations, panobinostat showed the best antileukemic activities and significantly enhanced cytarabine-induced apoptosis in AML cells, accompanied by cooperative induction of DNA DSBs [27]. Based on these new findings and previous studies that have shown panobinostat to be the most potent inhibitor among pan-HDACIs in clinical development [28], [29], we chose panobinostat as our prototype HDACI for this study.

The ability of HDACIs to enhance cytarabine-induced DNA DSBs and apoptosis in AML cells suggests that they may suppress the DNA damage response (DDR), a complex network involving cell cycle checkpoints, DNA repair, transcriptional programs, and apoptosis [30]–[33]. In cancer treatment, the DDR occurs in response to DNA damaging agents (e.g., cytarabine and DNR), representing an important mechanism limiting chemotherapeutic efficacy [30], [33]. BRCA1 and RAD51 are two of the central proteins in the homologous recombination DNA repair pathway [34]. Breast and ovarian cancer cells harboring *BRCA1* mutations are sensitive to DNA damaging agents and radiation therapy, highlighting the critical role of BRCA1 in response to DNA damaging agents [35], [36]. RAD51 expression was increased in a wide range of human tumors, most likely contributing to drug resistance [37]. Cell cycle checkpoint activation in response to DNA damage is another critical component of the DDR [30], [31]. CHK1 contributes to all currently defined cell cycle checkpoints [30]. It has been documented that inhibition of CHK1 with pharmacologic intervention or by siRNA knockdown sensitizes cancer cells to S/G2-phase-acting agents [30], [38]. Downregulation of BRCA1, CHK1, and RAD51 would enhance DNA damage and abrogate cell cycle checkpoints induced by DNA damaging agents, thus promoting apoptosis.

In this study, we found that panobinostat suppressed the expression of *BRCA1*, *CHK1*, and *RAD51* in AML cell lines and diagnostic blasts at clinically achievable doses (40 nM and below). This was accompanied by cooperative induction of DNA DSBs and apoptosis, and abrogation of cell cycle checkpoints induced by cytarabine or DNR. Analogous results were obtained by shRNA knockdown of BRCA1, CHK1, or RAD51 in AML cells. Collectively, our results strongly suggest that panobinostat suppresses the DDR, which represents a novel molecular mechanism underlying the antileukemic activities of HDACIs combined with DNA damaging agents in AML and potentially other cancers, as well.

## Materials and Methods

### Clinical Samples

Diagnostic bone marrow samples (n = 9, Table S1) from children with *de novo* AML were obtained from the Children’s Hospital of Michigan leukemia cell bank. Mononuclear cells were purified by standard Ficoll-Hypaque density centrifugation. Written informed consent was provided by the parent or legal guardian according to the Declaration of Helsinki. Sample handling and data analysis protocols were approved by the Human Investigation Committee of the Wayne State University School of Medicine.

### Drugs

Cytarabine and DNR were purchased from Sigma-Aldrich (St Louis, MO). Panobinostat was purchased from Selleck Chemicals (Houston, TX). Clinically, panobinostat steady-state plasma concentrations range from 15 to 22 nM over 48 h (data from Novartis Investigator’s Brochure).

### Cell Culture

THP-1 and U937 cell lines were purchased from the American Type Culture Collection (Manassas, VA). The OCI-AML3 cell line was purchased from the German Collection of Microorganisms and Cell Cultures (DSMZ, Braunschweig, Germany). The CTS cell line was a gift from Dr. A Fuse from the National Institute of Infectious Diseases, Tokyo, Japan [39]. The cell lines were cultured in RPMI 1640 (or αMEM for OCI-AML3 cells) media with 10% fetal bovine serum (Hyclone, Logan, UT) and 2 mM L-glutamine, plus 100 U/ml penicillin and 100 µg/ml streptomycin, in a 37°C humidified atmosphere containing 5% CO_2_/95% air.

### Panobinostat Treatment of Diagnostic Blast Samples

Diagnostic blast samples were cultured in RPMI 1640/20% dialyzed fetal bovine serum (Life Technologies, Carlsbad, CA) supplemented with ITS solution (Sigma-Aldrich) and 20% supernatant of the 5637 bladder cancer cell line (as a source of granulocyte-macrophage colony-stimulating factor) [40]. The cells were plated at a density of 1×10^6^ cells/mL and cultured in the presence of 0–40 nM panobinostat for 48 h in a 37°C humidified atmosphere containing 5% CO_2_/95% air.

### Assessment of Apoptosis

AML cell lines were treated with panobinostat and cytarabine or DNR for 48 h and subjected to flow cytometry analysis to determine drug-induced apoptosis using the Annexin V-fluorescein isothiocyanate (FITC)/propidium iodide (PI) Apoptosis Kit (Beckman Coulter; Brea, CA), as previously described [26], [27]. Results were expressed as percent of Annexin V+ cells. Synergy was quantified using the cooperativity index (cooperativity index = sum of apoptosis of single-agent treatment/apoptosis on combined treatment). Cooperativity index <1, 1, or >1 is termed synergistic, additive, or antagonistic, respectively [41]. Experiments were performed 3 independent times in triplicate. Data presented are from one representative experiment.

### Cell Cycle Progression

Cell cycle analysis was done as previously described [26]. Briefly, AML Cells were harvested and fixed with ice-cold 80% (v/v) ethanol for 24 h. After centrifugation at 200×g for 5 min, the cell pellets were washed with phosphate-buffered saline (PBS, pH 7.4) and resuspended in PBS containing PI (50 mg/mL), triton X-100 (0.1%, v/v), and DNase-free RNase (1 mg/mL). The DNA contents were determined by flow cytometry using a FACScan flow cytometer (BD Biosciences, San Jose, CA). Data analysis was done with the Multicycle software (Phoenix Flow Systems, Inc., San Diego, CA). Experiments were performed 3 independent times in triplicate. Data presented are from one representative experiment.

### Quantification of Gene Expression by Real-time RT-PCR

Total RNA was extracted using TRIzol (Life Technologies) and cDNAs were prepared from 1 µg total RNA using random hexamer primers and a RT-PCR kit (Life Technologies), and purified with the QIAquick PCR Purification Kit (Qiagen, Valencia, CA) as previously described [27], [42]. *BRCA1* (Hs00173237_m1), *CHK1* (Hx00967506_m1), *RAD51* (Hs00153418_m1) *and E2F1* (Hs00153451_m1) transcripts were quantitated using Taqman probes (Life Technologies) and a LightCycler real-time PCR machine (Roche Diagnostics, Indianapolis, IN), based on the manufacturer’s instructions. Real-time PCR results were expressed as means from 3 independent experiments and were normalized to *GAPDH* (4333764) or *RPL13a* (Hs03043885_g1) transcripts. Fold changes were calculated using the comparative *C_t_* method [43].

### Western Blot Analysis

Cells were lysed in Tris buffer (10 mM, pH 8.0) containing protease inhibitors (Roche Diagnostics). Whole cell lysates were subjected to SDS-polyacrylamide gel electrophoresis, electrophoretically transferred onto polyvinylidene difluoride (PVDF) membranes (Thermo Fisher Inc., Rockford, IL) and immunoblotted with anti-acetyl-histone 4 (ac-H4, 06–598), -H4 (07–108) (Upstate Biotechnology, Lake Placid, NY), -BRCA1 (9010), -γH2AX (2577), -RPL13a (2765), -Caspase 3 (9661), -PARP (9542), -p-CDC25C (9528), -E2F1 (3742) (Cell Signaling Technology, Danvers, MA), -RAD51 (sc-8349), -CHK1 (sc-8408, Santa Cruz Biotechnology, Santa Cruz, CA) -acetyl-tubulin (T7451) or -β-actin (A2228, Sigma-Aldrich) antibody, as described previously [42]. Immunoreactive proteins were visualized using the Odyssey Infrared Imaging System (Li-Cor, Lincoln, NE), as described by the manufacturer. Western blots were repeated at least 3 times and one representative blot is shown.

### In Vitro Cytotoxicity Assays

*In vitro* cytotoxicities of AML diagnostic blasts were measured by using MTT (3-[4,5-dimethyl-thiazol-2-yl]-2,5-diphenyltetrazoliumbromide, Sigma-Aldrich) assays, as previously described [44]. Briefly, the cells were cultured as mentioned in “Panobinostat Treatment of Diagnostic Blast Samples” and treated with variable concentrations of panobinostat (0–160 nM) for 48 hours. IC_50_ values were calculated as drug concentrations necessary to inhibit 50% proliferation compared to untreated control cells. IC_50_ values are means of duplicates from one experiment.

### Production of Lentivirus Particles and Transduction of THP-1 Cells

The pMD-VSV-G and delta 8.2 plasmids were gifts from Dr. Dong at the Tulane University. The transfection was carried out by using Lipofectamine and Plus reagents (Life Technologies) according to the manufacturer’s instructions. Briefly, a lentivirus vector (BRCA1, CHK1, or RAD51 shRNA construct from Sigma-Aldrich or CHK1 cDNA construct from Thermo Fisher Scientific Biosciences, Lafayette, CO), pMD-VSV-G and delta 8.2 were cotransfected into TLA-HEK293T cells and the culture medium was harvested 48 h post-transfection. One million THP-1 Cells were transduced by adding 1 ml of virus supernatant and 4 µg of polybrene (Sigma-Aldrich) [26], [27], [42].

#### Comet assay

THP-1 cells, infected with NTC-, BRCA1-, CHK1- or RAD51-shRNA, were treated with 50 µM cytarabine for 3 h. The cells were washed and cultured for up to 8 h. The harvested cells were mixed with 1.0% low melting point agarose in PBS (Mg and Ca free) at 37^o^C. Approximately 5000 cells were layered onto pre-coated microscope slides (pre-coated slides were coated with a thin layer of 1% low melting point agarose and allowed to dry for 90 min), a glass coverslip was placed on top of the cell/agarose suspension and the gels were allowed to gel for 10 min at 4°C. The slides were then placed in lysis solution (2.5 M NaCl, 10 mM Tris, 100 mM EDTA, 10% DMSO and 1% Triton X-100, pH 10.0) overnight at 4°C. The slides were placed in electrophoresis buffer (1 mM EDTA, 300 mM NaOH, pH 13.0) for 40 min at 4°C to allow unwinding of the DNA. Electrophoresis was conducted for 30 min at 30 V (1.25 V/cm). After electrophoresis, the slides were rinsed in 400 mL distilled water, dipped in 95% ethanol and dried. DNA was stained with 1∶30,000 SYBR Gold (Life Techonologies) in 10 mM Tris, pH 7.5 plus 1 mM EDTA for 40 min at room temperature. The slides were rinsed in distilled water and imaged on an Olympus BX-40 microscope with an Olympus DP72 microscope camera and Olympus cellSens Dimension software (Olympus America Inc., Center Valley, PA). 50 comets per gel were scored using CometScore (TriTek Corp, Sumerduck, VA). The median percent DNA in the tail from at least three replicate gels were averaged and graphed. The error bars indicate standard errors of the means.

### Leukemia Xenograft Model

U937 cells were transfected with a murine stem cell virus-green fluorescent protein vector containing the firefly Luciferase gene (pMSCV-luc-IRES-GFP), sorted by flow cytometry, expanded in culture and 10,000 cells were injected intravenously into the tail vein of 8- to 12-week-old female NOD-SCID-IL2Rγ^null^ (NSG) mice (n = 36), as described previously [45].

To evaluate antileukemic activity, groups of 8–10 tumor-bearing mice were randomly assigned to receive panobinostat [5 mg/kg once daily for 3 weeks, provided by Novartis Pharma AG (Basel, Switzerland)], cytarabine (6.25 mg/kg once daily for 4 weeks), or combination (panobinostat 5 mg/kg once daily for 3 weeks plus ara-C 6.25 mg/kg once daily for 4 weeks) starting on day 3 after injection of U937 cells. A group of vehicle treated mice were included as controls with each experiment. For intraperitoneal injection panobinostat was formulated in 5% dextrose and cytarabine was formulated in PBS.

Noninvasive imaging was performed twice weekly in all experiments to monitor tumor engraftment, as previously described [45]. The Luciferase substrate D-luciferin firefly potassium salt (Caliper, Hopkinton, MA) at a dose of 150 mg/kg was administered by intraperitoneal injection. The mice were then anesthetized by 1.5%–2.5% isoflurane inhalation, and bioluminescence was done 5 minutes later using a Xenogen in vivo imaging system (Hopkinton, MA) in the Animal Imaging Facility at St. Jude Children’s Research Hospital. Total bioluminescence was quantified for the body area that included each mouse in its entirety. Mice were monitored daily and were sacrificed by CO_2_ asphyxiation when they showed signs of terminal illness, including hind limb paralysis, inability to eat or drink, and/or moribund. All animal experiments were approved by the Institutional Animal Care and Use Committee of St. Jude Children’s Research Hospital.

Pharmacodynamic (PD) studies were performed to determine *in vivo* effects of panobinostat treatment on BRCA1, CHK1 and RAD51. When bioluminescence reached 1.5×10^7^ p/s/cm^2^/sr, day 17 after injection of U937 cells, mice received panobinostat (5 mg/kg once daily×2). Four hours after the second dose mice were sacrificed, bone marrow was harvested, and snap frozen pellets were stored at −80°C. The percentage of leukemic cell infiltration in bone marrow was determined by hematoxylin and eosin staining.

### Chromatin Immunoprecipitation Assay

The chromatin immunoprecipitation (ChIP) assay was performed as described previously [42]. THP-1 cells were treated with or without 10 nM panobinostat for 48 h. Anti-E2F1 antibody (sc-193x) or normal rabbit IgG (sc-2027, Santa Cruz Biotechnology) were used for the immunoprecipitation. Real-time PCR for the E2F1 binding regions in the *BRCA1, CHK1, or RAD51* promoter was performed using the primers listed in Table S2.

### Statistical Analysis

Differences in cell apoptosis and differences in transcript levels between cytarabine/DNR and panobinostat treated (individually or combined) and untreated cells were compared using the pair-wise two-sample t-test. The Kaplan-Meier method was used to estimate survival probability. Statistical analyses were performed with GraphPad Prism software. Error bars on all bar graphs represent standard errors of the means.

## Results

### Panobinostat Suppresses BRCA1, CHK1, and RAD51 Expression in AML Cell Lines and Diagnostic Blasts

**Figure 1:**
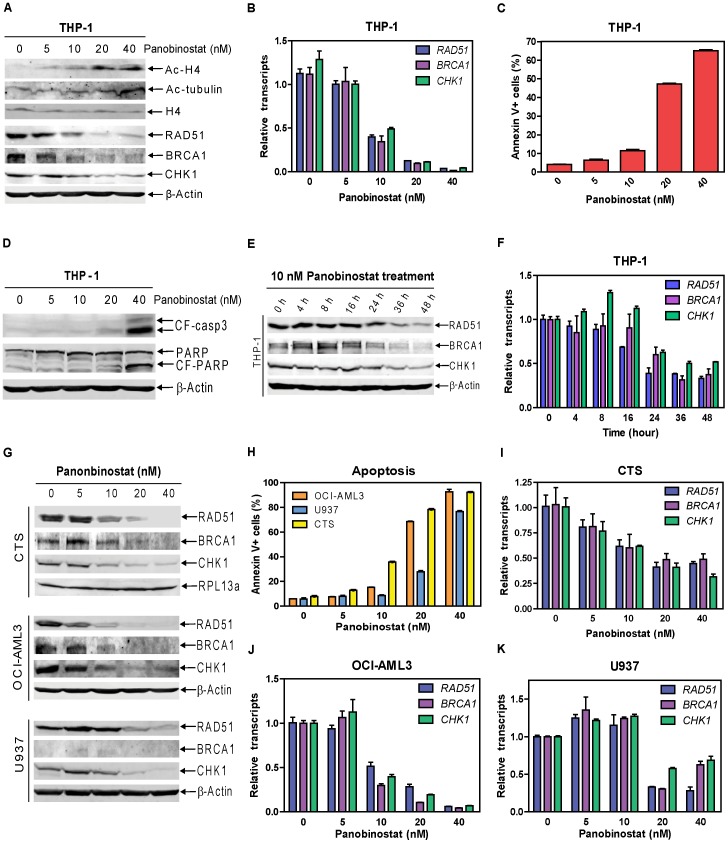
Panobinostat suppresses *BRCA1*, *CHK1*, and *RAD51* protein and transcript expression and induces apoptosis in AML cell lines. THP-1 cells were treated with variable concentrations of panobinostat for 48 h. Whole cell lysates were subjected to Western blotting (**Panels A&D**). Total RNAs were isolated and mRNA levels were determined by Real-time RT-PCR (**Panel B**). Apoptotic events were determined by annexin V/PI staining and flow cytometry analysis (**Panel C**). Protein and mRNA levels for *BRCA1*, *CHK1*, and *RAD51* genes were determined by Western blotting (**Panel E**) and Real-time PCR (**Panel F**), respectively, in THP-1 cells treated with 10 nM panobinostat for up to 48 h. CTS, OCI-AML3 or U937 AML cells were treated with variable concentrations of panobinostat for 48 h. Whole cell lysates were subjected to Western blotting to measure protein levels for BRCA1, CHK1, and RAD51 in the cells (**Panel G**). The levels of apoptosis induced by panobinostat were determined by flow cytometry analysis with annexin V/PI staining (**Panel H**). Transcript levels for *BRCA1*, *CHK1*, and *RAD51* genes were determined by Real-time RT-PCR (**Panels I–K**).

To test our hypothesis that HDACIs suppress expression of critical DDR genes in AML cells, we used 0–40 nM panobinostat to treat THP-1 cells for 48 h and determined protein and transcript levels for various DDR genes. Treatments resulted in hyperacetylation of histone H4 in a dose-dependent manner, while having no effect on total histone H4 levels. Treatment with the highest dose also caused hyperacetylation of alpha-tubulin (Figure 1A). Panobinostat suppressed BRCA1, CHK1, and RAD51 in a dose-dependent manner (Figure 1A), accompanied by dose-dependent downregulation of transcripts for the corresponding genes (by real-time RT-PCR), suggesting a possible transcriptional mechanism (Figure 1B). This correlates with dose-dependent induction of apoptosis and the cleavage of caspase 3 and PARP (Figures 1C&D). In contrast, other critical DDR proteins, such as MRE11, RAD50, 53BP1, ATM, ATR, Ku70, and CHK2, were not altered (data not shown). Panobinostat suppressed BRCA1, CHK1, and RAD51 expression and induced minimal apoptosis at 10 nM (Figures 1A&C). A time course experiment showed that 10 nM panobinostat required 36–48 h to maximally suppress the expression of *BRCA1*, *CHK1*, and *RAD51* (Figures 1E&F). Dose-dependent suppression of *BRCA1*, *CHK1*, and *RAD51* transcript and protein expression was also detected in CTS, U937, and OCI-AML3 AML cell lines (Figures 1G&I–K, BRCA1 was barely detectable in the U937 cells, and thus drug effects are difficult to interpret). This was accompanied by dose-dependent induction of apoptosis (Figure 1H).

To further confirm these results, we treated nine diagnostic AML blast samples with varying doses of panobinostat. Decreased transcript levels for *BRCA1*, *CHK1*, and *RAD51* genes were detected by real-time RT-PCR. This was accompanied by dose-dependent growth arrest, determined by MTT assays (Table 1). Together, these results demonstrate that panobinostat suppresses expression of *BRCA1*, *CHK1*, and *RAD51* genes in AML cell lines and decreases transcript levels in diagnostic AML blasts.

**Table 1:** Effects of panobinostat treatment on the transcript levels for *BRCA1*, *CHK1*, and *RAD51* genes in diagnostic AML blasts.

Patient	panobinostat	Relative transcripts post panobinostat treatments (0–40 nM for 48 h)												
sample	IC_50_ (nM)	*BRCA1*				*CHK1*				*RAD51*				
		0	10	20	40	0	10	20	40		0	10	20	40
A30074	57.8	1.04±0.16	1.09±0.07	0.47±0.04	0.03±0.01	1.02±0.12	1.30±0.10	0.76±0.04	0.08±0.01		1.04±0.15	1.11±0.13	0.57±0.07	0.06±0.00
A30310	98.4	1.01±0.06	0.32±0.02	0.37±0.06	0.17±0.02	1.03±0.11	0.36±0.03	0.43±0.05	0.20±0.02		1.04±0.17	0.29±0.03	0.38±0.05	0.24±0.03
A30320	61.3	1.02±0.10	0.12±0.02	0.11±0.04	0.03±0.01	1.01±0.08	0.11±0.02	0.05±0.02	0.05±0.01		1.03±0.14	0.11±0.02	0.05±0.01	0.03±0.00
A30321	34.1	1.04±0.16	0.16±0.02	0.09±0.01	0.03±0.01	1.02±0.12	0.20±0.02	0.16±0.01	0.05±0.02		1.06±0.20	0.12±0.01	0.11±0.01	0.02±0.01
A30322	38.1	1.02±0.09	0.73±0.15	0.55±0.05	0.13±0.03	1.01±0.10	0.76±0.11	0.59±0.11	0.13±0.02		1.02±0.11	0.94±0.26	0.62±0.13	0.11±0.03
A30323	41.2	1.01±0.10	0.53±0.07	0.25±0.04	0.11±0.01	1.02±0.11	0.73±0.11	0.49±0.03	0.31±0.02		1.06±0.21	0.75±0.16	0.32±0.02	0.21±0.03
A30326	55.5	1.01±0.06	0.51±0.03	0.19±0.02	0.04±0.01	1.01±0.09	0.65±0.04	0.22±0.03	0.09±0.01		1.02±0.11	0.71±0.10	0.25±0.04	0.13±0.01
A30329	10	1.02±0.12	0.11±0.02	0.04±0.01	0.02±0.01	1.03±0.15	0.11±0.01	0.06±0.01	0.03±0.01		1.05±0.20	0.10±0.02	0.04±0.01	0.03±0.01
A30338	46.7	1.00±0.05	0.50±0.09	0.29±0.05	0.10±0.03	1.01±0.10	0.55±0.06	0.40±0.07	0.19±0.05		1.03±0.15	0.53±0.11	0.44±0.07	0.09±0.03

Note: Transcript levels for *BRCA1*, *CHK1*, and *RAD51* in panobinostat treated and untreated cells were quantified by real-time RT-PCR and normalized to transcript levels of *RPL13A*. Results are expressed as means of three independent experiments relative to that of the untreated cells (set as 1).

### Panobinostat Cooperates with Cytarabine or DNR in Inducing DNA DSBs and Apoptosis in AML Cells

**Figure 2:**
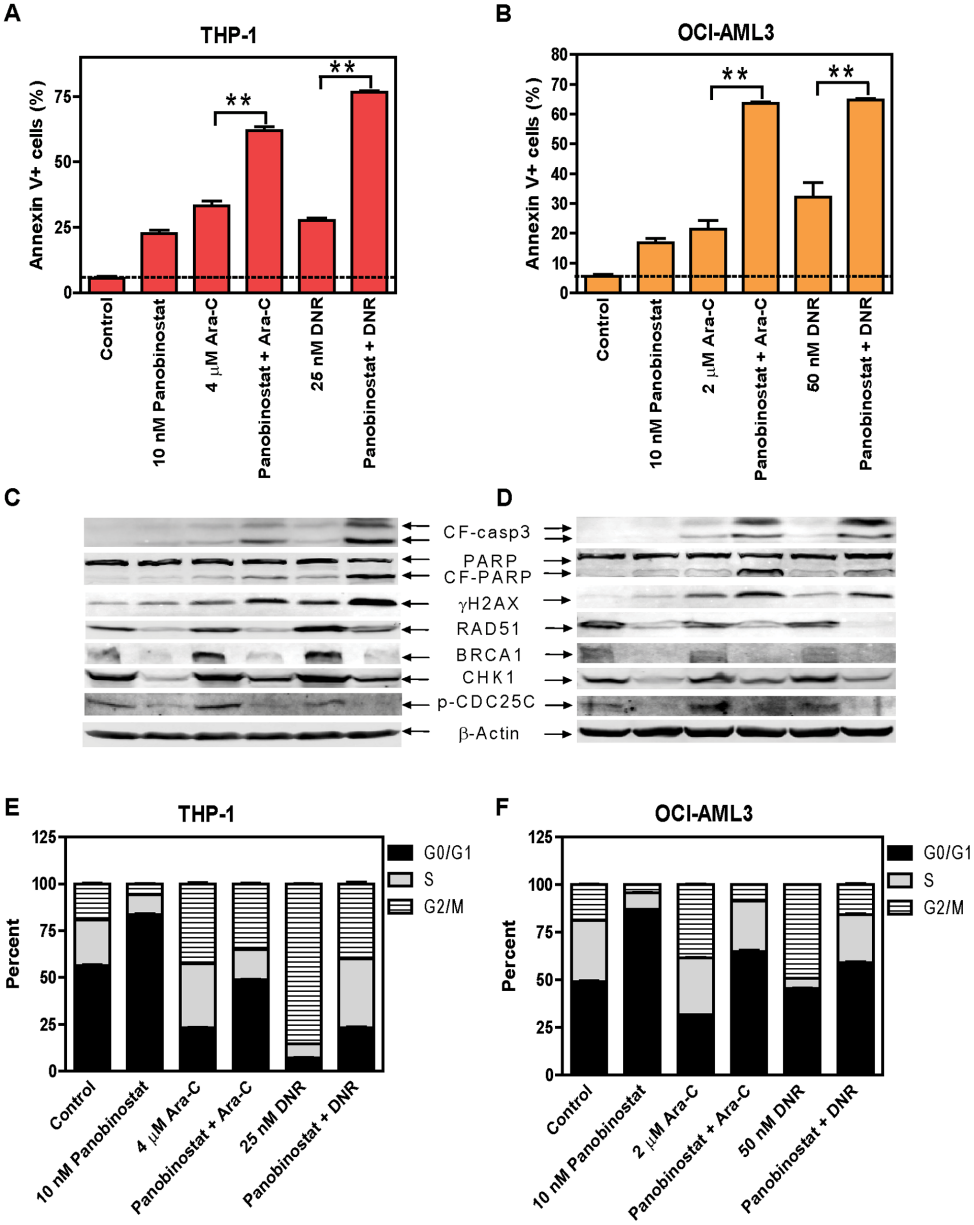
Panobinostat cooperates with cytarabine or DNR in inducing DNA DSBs and apoptosis, and abrogates S and/or G2/M cell cycle checkpoint activation induced by cytarabine or DNR in THP-1 and OCI-AML3 AML cells. THP-1 or OCI-AML3 cells were treated with cytarabine or DNR, alone or in combination with panobinostat for 48 h. Early and late apoptosis events in the cells were determined by annexin V/PI staining and flow cytometry analyses (**Panels A&B**). Whole cell lysates were subjected to Western blotting (**Panels C&D**). Cell cycle distribution was determined by PI staining and flow cytometry analysis (**Panels E&F**). **indicates p<0.005.

Efforts were then undertaken to determine the effects of panobinostat on cytarabine- or DNR-induced DNA DSBs, cell cycle progression and apoptosis by treating THP-1 or OCI-AML3 cells for 48 h. Cytarabine- or DNR-induced apoptosis was significantly enhanced by the addition of panobinostat (Figures 2A–D). Representative dot plots for the THP-1 cell line can be found in Figure S1A–F. Combined cytarabine and panobinostat treatment caused synergistic induction of apoptosis with cooperativity index values of 0.87 and 0.55 for THP-1 and OCI-AML3 cells, respectively. DNR and panobinostat also caused synergistic induction of apoptosis with cooperative index values of 0.63 and 0.67 for THP-1 and OCI-AML3 cells, respectively. Co-treated THP-1 and OCI-AML3 cells had reduced protein levels for BRCA1, CHK1, and RAD51 compared to cells treated with cytarabine or daunorubicin alone. Downregulation of CHK1 decreased activation of the pathway, as reflected by the decreased phosphorylation of CDC25C (Ser 216, Figures 2C&D). Importantly, panobinostat substantially enhanced cytarabine- or DNR-induced DNA DSBs, as reflected by the induction of γH2AX, an established biomarker for DNA DSBs [46] (Figures 2C&D). Essentially the same results were obtained in U937 cells (Figures S2A&C). Although CTS cells did not display the same cooperative induction of apoptosis by panobinostat and cytarabine, DNR and panobinostat did (Figures S2B&D).

Panobinostat treatment alone resulted in G0/G1 arrest in THP-1 and OCI-AML3 cells. Cytarabine treatment resulted in S and G2/M arrest in both cell lines, which were decreased with the addition of panobinostat. DNR treatment resulted in G2/M arrest, which was reduced by the co-administration with panobinostat (Figures 2E&F). Representative histograms for the THP-1 cell line can be found in Figure S1G–L. Similar results were obtained in U937 cells treated with panobinostat and cytarabine or DNR and in CTS cells treated with panobinostat and DNR (Figures S2E&F). However, CTS cells treated with cytarabine and panobinostat resulted in increased S and G2/M, which parallels with modestly enhanced apoptosis compared to that from cytarabine alone (Figure S2F). These results suggest that at least partial abrogation of the S and/or G2/M cell cycle checkpoints is required for the cooperative induction of apoptosis by panobinostat and cytarabine or DNR in AML cells.

### The Roles of BRCA1, CHK1, and RAD51 in DNA DSBs and Apoptosis Induced by Cytarabine or DNR in AML Cells

**Figure 3:**
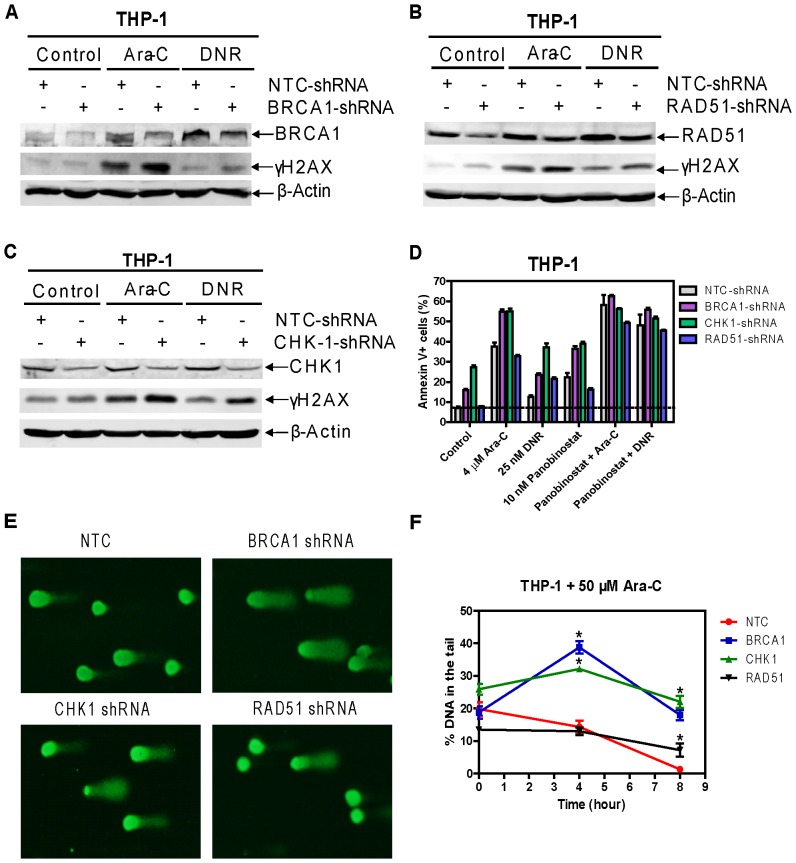
The roles of BRCA1, CHK1, and RAD51 in cytarabine- or DNR-induced DNA DSBs and apoptosis in THP-1 cells. THP-1 cells were infected with BRCA1, CHK1, RAD51, or non-target control (NTC) shRNA lentivirus overnight, washed and then treated with 4 µM cytarabine or 25 nM DNR for 48 h. shRNA knockdown of BRCA1, CHK1, or RAD51, and induction of γH2AX by cytarabine or DNR were determined by Western blotting (**Panels A–C**). The lane headings indicate the treatment conditions ‘Control’ ‘Ara-C’ or ‘DNR’ and the+or – indicate the shRNA-treated cells from which the sample was derived. Apoptotic events in the cells were determined by annexin V/PI staining and flow cytometry analyses (**Panel D**). THP-1 cells were infected with BRCA1, CHK1, or RAD51 shRNA lentivirus overnight. The cells were washed three times with complete medium and cultured in virus-free complete medium for up to 72 h. The cells were then treated with 50 µM cytarabine or 2 µM DNR for 3 h and the drugs were washed out, and the cells were cultured in drug-free complete medium for up to 8 h. DNA damage was assessed by COMET assay. Representative images at the 8 h time point are shown (**Panel E**). The median percent DNA in the tail from at least three replicate gels are shown plus or minus the standard error of the mean (Panel F). *indicates p<0.05.

To provide direct evidence that BRCA1, CHK1, and RAD51 were critical for the cooperative antileukemic activities of cytarabine or DNR with panobinostat, lentivirus shRNA knockdown of each gene was performed in THP-1 cells. *BRCA1* shRNA knockdown cells (designated *BRCA1*-shRNA cells) treated with cytarabine, and to a lesser extent DNR, displayed higher levels of DNA DSBs as measured by phosphorylation of H2AX compared to controls cells (designated NTC-shRNA, Figure 3A). Significantly increased basal and cytarabine-, DNR-, or panobinostat-induced apoptosis was detected in the *BRCA1*-shRNA cells compared to the NTC-shRNA cells (Figure 3D). Further, shRNA knockdown of *BRCA1* almost completely abolished panobinostat enhancement on cytarabine-induced apoptosis, however, its effect on DNR was much less pronounced (Figure 3D). shRNA knockdown of *BRCA1* partially abrogated cytarabine-induced S checkpoint and DNR-induced G2/M checkpoint (Figures S3A&B). shRNA knockdown of *RAD51* displayed increased DNA DSBs when compared to the NTC-shRNA control cells (Figure 3B). It also showed enhanced DNR-induced apoptosis, whereas it had no impact on basal and cytarabine-induced apoptosis, and S or G2/M checkpoints induced by cytarabine or DNR (Figures 3B&D, S3C&D). The *CHK1* shRNA knockdown resulted in significantly increased basal and cytarabine- or DNR-induced apoptosis, along with substantially increased DSBs and abrogated S and G2/M checkpoints (Figures 3C&D, S3E&F). Alternately, ectopic overexpression of CHK1 resulted in significantly decreased apoptosis induced by panobinostat alone or its combination with cytarabine or DNR, but had no impact on apoptosis induced by cytarabine or DNR alone (Figures S4A&B). This was accompanied by loss of effectiveness of panobinostat on the ectopically expressed CHK1 protein (Figures S4C&D). These results provide strong evidence that BRCA1 and CHK1, and to a lesser extent RAD51, are critical mediators of the cooperative antileukemic activities of combined panobinostat and cytarabine or DNR in AML cells.

To confirm that BRCA1, CHK1, or RAD51 knockdown had an impact on DNA damage after cytarabine treatment, shRNA knockdown cells were treated for three hours with 50 µM cytarabine followed by washout. DNA damage was then directly measured using the comet assay at 0, 4, and 8 hours post-washout. As can be seen in Figure 3E&F, BRCA1-and CHK1-shRNA cells had higher levels of damage at 4 hours when compared to the NTC-shRNA cells. BRCA1-, CHK1-, and RAD51-shRNA cells all had higher damage at 8 hours when compared to NTC-shRNA cells.

### Antitumor Activity of Panobinostat Combined With Cytarabine in a Xenograft Model of AML

**Figure 4:**
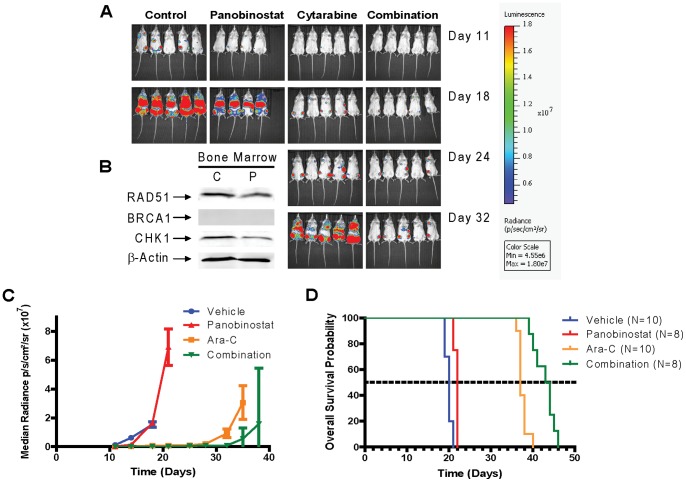
Antileukemic activity of panobinostat alone, cytarabine alone, and panobinostat plus cytarabine in a U937 xenograft model. NOD-SCID-IL2Rγ^null^ (NSG) mice were injected with luciferase-labeled U937 cells and treated 3 days later with panobinostat (5 mg/kg once daily for 3 weeks), ara-C (6.25 mg/kg once daily for 4 weeks), or combination (panobinostat 5 mg/kg once daily for 3 weeks plus ara-C 6.25 mg/kg once daily for 4 weeks). Serial bioluminescence images of representative mice receiving panobinostat alone (n = 8), ara-C alone (n = 10), or panobinostat plus ara-C (n = 8) (**Panel A**). When bioluminescence reached 1.5×10^7^ p/s/cm^2^/sr, day 17 after injection of U937 cells, mice received panobinostat (5 mg/kg once daily×2). Four hours after the second dose mice were sacrificed and Bone Marrows were harvested. Pellets were lysed and subjected to Western Blot (**Panel B**). Tumor progression monitored by quantitative biophotonic imaging analysis of control and treatment groups (**Panel C**). A plot of overall survival probability, estimated with the Kaplan–Meier method (**Panel D**).

The antileukemic activity of cytarabine and panobinostat alone or in combination compared with vehicle treated controls was evaluated in NSG mice engrafted with the U937 cells. Differences in tumor engraftment and progression were monitored by bioluminescence imaging (Figure 4A) and quantitative biophotonic imaging analysis (Figure 4C), and the incidence of death due to leukemia was calculated (Figure 4D). After panobinostat treatment, downregulation of RAD51 and CHK1 protein expression was observed in bone marrow samples with ≥60% leukemic cell infiltration (Figure 4B). The expression of BRCA1 in these samples was barely detectable (Figure 4B) analogous to results from *in vitro* studies (Figure 1G).

Death due to leukemia progression among vehicle or panobinostat treated mice was similar (20 vs 22 days, respectively). In accordance with previous studies, tumor progression was quantified by assessment of the increase in median bioluminescence signal [45]. On day 18 there was a statistically significant delay in tumor progression by cytarabine-treated mice compared with vehicle controls (cytarabine alone vs control, median = 7.62×10^5^ photon/s/cm^2^/sr vs controls = 1.54×10^7^ photon/s/cm^2^/sr, p = 0.002). By day 32, significant differences in tumor progression were observed between mice treated with panobinostat daily plus cytarabine compared to cytarabine (panobinostat plus cytarabine vs cytarabine, median = 7.78×10^5^ photon/s/cm^2^/sr vs cytarabine = 9.19×10^6^ photon/s/cm^2^/sr, p = 0.0078). By pair-wise comparisons, administration of panobinostat combined with cytarabine significantly prolonged median survival compared with cytarabine alone (p = 0.0001), panobinostat alone (p = 0.0002), and the control (p<0.0001) (panobinostat plus cytarabine, median survival = 44 days; cytarabine alone, median survival = 37 days; panobinostat, median survival = 22 days; and control, median survival = 20 days; Table S3).

### E2F1 Is a Critical Mediator of the Suppression of BRCA1, CHK1, and RAD51 Expression by Panobinostat in AML Cells

**Figure 5:**
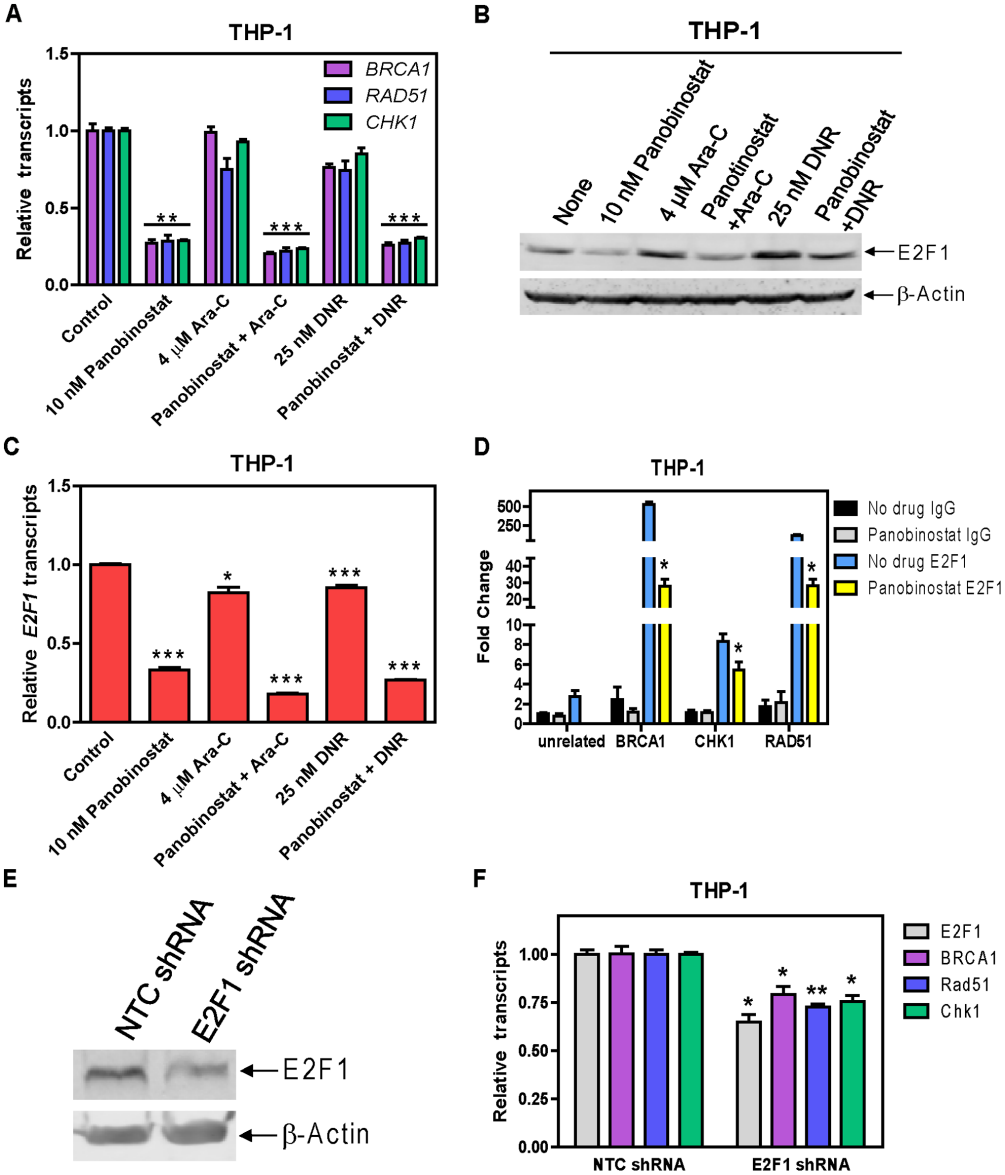
E2F1 is a critical mediator of the suppression of BRAC1, CHK1, and RAD51 by panobinostat in THP-1 cells. THP-1 cells were treated with cytarabine or DNR alone or in combination with panobinostat for 48 h. Transcript levels for *BRCA1*, *CHK1*, *RAD51,* and *E2F1* genes were determined by Real-time RT-PCR (**Panels A&C**). Whole cell lysates were subjected to Western blotting and probed with E2F1 antibody (**Panel B**). *In vivo* binding of E2F1 to the putative binding sites located in the *BRCA1*, *CHK1*, or *RAD51* promoter was determined by ChIP assays with or without 10 nM panobinostat treatment for 48 h with the use of real-time PCR as described in “Material and Methods” (**Panel D**). THP-1 cells were infected with E2F1 or non-target control (NTC) shRNA lentivirus overnight, washed and cultured for another 72 h. shRNA knockdown of E2F1 was determined by Western blotting (**Panel E**) and real-time RT-PCR (**Panel F**). Effects of E2F1 knockdown on the transcript levels for *BRCA1*, *CHK1*, or *RAD51* were determined by real-time RT-PCR (**Panel F**). *indicates p<0.05, **indicates p<0.005, ***indicates p<0.0005.

To begin to understand the molecular mechanism responsible for the suppression of BRCA1, CHK1, and RAD51 expression by panobinostat, transcript levels of *BRCA1, CHK1,* and *RAD51* were determined in THP-1 cells following 48 h drug treatments. Panobinostat treated cells had decreased transcript levels of these genes independent of cytarabine or DNR, while cytarabine or DNR alone had little to no effect on the transcript levels of these genes (Figure 5A). These results suggest that panobinostat suppresses transcription of *BRCA1*, *CHK1*, and *RAD51* genes in AML cells.

Based on the reported roles of E2F1 in the DDR and in the transcriptional regulation of *BRCA1, CHK1,* and *RAD51* [47]–[50], we hypothesized that E2F1 is a critical mediator of the suppression of these genes by panobinostat. Treatments of THP-1 cells with cytarabine or DNR resulted in higher E2F1 protein levels and a small decrease in E2F1 transcript levels. Administration of panobinostat suppressed expression of E2F1 protein and transcripts independent of cytarabine or DNR (Figure 5B&C). This was accompanied by significantly decreased binding of E2F1 to the promoter regions of *BRCA1*, *CHK1*, and *RAD51* determined by ChIP assays (Figure 5D). These results strongly suggest that panobinostat suppresses expression of *BRCA1, CHK1,* and *RAD51* through modulation of E2F1 transcription factor.

To provide direct evidence that E2F1 regulates the transcription of these genes in AML cells, we performed shRNA knockdown of E2F1 in THP-1 cells (Figures 5E&F). Knockdown of E2F1 resulted in significantly decreased transcript levels for *BRCA1, CHK1,* and *RAD51*. These results provide compelling evidence that panobinostat suppresses expression of *BRCA1, CHK1*, and *RAD51* by transcriptionally downregulating the expression of E2F1.

## Discussion

In previous studies, we demonstrated synergistic antileukemic activities of combined VPA and cytarabine or clofarabine in pediatric AMLs accompanied by cooperative induction of DNA DSBs and apoptosis [26], [51]. This observation was also confirmed with other HDACIs, such as panobinostat, when combined with cytarabine in AML cells [27]. However, the molecular mechanisms underlying the cooperative induction of DNA DSBs and apoptosis remained unknown. The abilities of different HDACIs to enhance cytarabine-induced DNA DSBs suggest that this class of drugs may somehow impact the DDR to enhance DNA DSBs and apoptosis induced by DNA damaging agents in AML cells.

To test this possibility, we determined the effects of panobinostat treatments on the expression of critical DDR proteins in four AML cell lines and nine pediatric diagnostic AML blast samples. Though the use of only pediatric samples in this study is an admitted weakness, *in vitro* incubations of these AML cells with panobinostat demonstrated dose-dependent decreases in protein and/or transcript levels for *BRCA1*, *CHK1*, and *RAD51* genes (Figure 1 and Table 1), suggesting that panobinostat suppresses the DDR in AML cells by decreasing *BRCA1, CHK1,* and *RAD51* transcript levels.

Consistent with the above findings, panobinostat decreases the expression of BRCA1, CHK1, and RAD51, potently enhanced DNA DSBs and apoptosis, and abrogated S and/or G2/M checkpoints induced by cytarabine or DNR in 3 out of the four AML cell lines (THP-1, U937, and OCI-AML3, Figures 2 and S2A, C, and E). In CTS cells these changes were observed in the DNR and panobinostat co-treatment (Figures S2B, D and F). Although the downregulation of BRCA1, CHK1, and RAD51 protein expression was also observed in the CTS cells post combined treatment with panobinostat and cytarabine (Figure S2D), more S and G2/M arrest were observed. It has been reported that a CHK1-independent S checkpoint pathway exists and parallels the CHK1-dependent S checkpoint pathway [52]. This may explain the observed activation of the S checkpoint despite the decreased level of BRCA1 and CHK1 and the modest enhancement of panobinostat on cytarabine-induced apoptosis in the CTS cells. These results demonstrate that the enhanced antileukemic activities of cytarabine or DNR by panobinostat rely on abrogation of the S and/or G2/M cell cycle checkpoints induced by cytarabine or DNR.

Our *BRCA1, CHK1*, and *RAD51* shRNA knockdown results in THP-1 cells provide direct evidence that these proteins play critical roles in cytarabine- or DNR-induced DNA damage, which was confirmed by our COMET assay experiment (Figure 3). Flow cytometry analysis revealed that both BRCA1 and CHK1 are critical determinants of both cytarabine- and DNR-induced apoptosis in AML cells as indicated by the enhanced apoptosis seen in the knockdown cells after single drug treatment, while the effects of RAD51 knockdown seem DNR-specific since it only enhanced DNR-induced apoptosis (Figure 3). Interestingly, both BRCA1 and CHK1, but not RAD51, are involved in the S and/or G2/M checkpoints induced by cytarabine or DNR (Figure S3). These results are consistent with panobinostat and cytarabine or DNR co-treatments and strongly demonstrate that suppression of the DDR by panobinostat represents a novel and critical molecular mechanism underlying the cooperative induction of DNA DSBs and apoptosis by the combination of panobinostat and cytarabine or DNR. The role of CHK1 in the combined antileukemic activities of panobinostat and cytarabine or DNR was further confirmed by ectopically expressing it in THP-1 cells (Figure S4). *In vivo* NSG mouse studies showed decreased levels of CHK1 and RAD51 post panobinostat treatments. In addition, co-treatment of the tumor-bearing NSG mice with panobinostat and cytarabine resulted in significant delay of tumor growth and significantly increased survival compared to cytarabine or panobinostat treatment alone (Figure 4 and Table S3).

Results from our additional studies suggest that panobinostat suppresses the expression of BRCA1, CHK1, and RAD51 through transcriptional mechanisms, in which E2F1 plays a critical role (Figure 5), however, post-transcriptional mechanisms cannot be completely excluded. Studies are underway investigating the molecular mechanisms by which panobinostat regulates E2F1 expression in AML cells.

**Figure 6:**
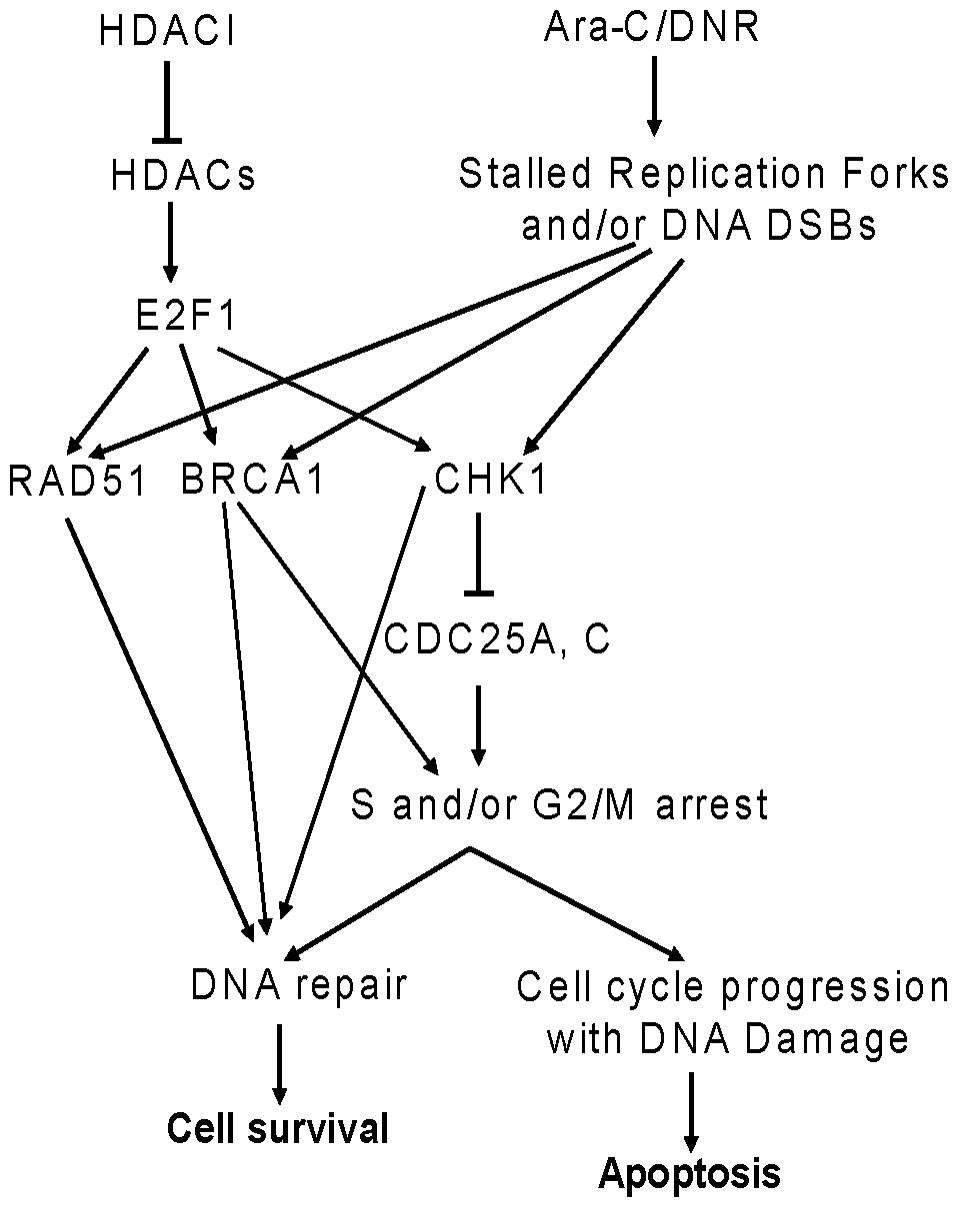
A proposed model of molecular mechanisms underlying the cooperative induction of apoptosis by HDACIs and DNA damaging agents in AML cells. HDACIs suppress the transcript and protein levels of the transcription factor gene *E2F1*, leading to decreased expression of BRCA1, CHK1, and RAD51. Decreased expression of BRCA1, CHK1, and RAD51 would result in weakened repair of DNA DSBs induced by DNA damaging chemotherapeutic agents (e.g., cytarabine or DNR), thus enhancing apoptosis. At the same time, downregulation of BRCA1 and CHK1 by HDACIs would result in abrogation of cell cycle checkpoint activation (S and/or G2/M) induced by DNA damaging agents, thus forcing cells carrying DNA lesions to progress in the cell cycle and undergo apoptosis.

Based on our own results and those previously reported [30], we have proposed a model for the molecular mechanisms underlying the cooperative induction of apoptosis by HDACIs and DNA damaging agents in AML cells (Figure 6). HDACIs suppress the expression of E2F1, leading to decreased E2F1 binding to the *BRCA1, CHK1,* and *RAD51* promoters and decreased transcription of these genes. This would result in weakened repair of DNA DSBs induced by DNA damaging chemotherapeutic agents (e.g., cytarabine or DNR), thus enhancing apoptosis. At the same time, downregulation of BRCA1 and CHK1 would result in abrogation of cell cycle checkpoints (S and/or G2/M), thus forcing cells carrying DNA lesions to progress in the cell cycle and undergo apoptosis.

In summary, our study demonstrates that panobinostat suppresses the expression of *BRCA1, CHK1*, and *RAD51 in vitro* and *in vivo*, leading to enhancement of DNA damage, abrogation of cell cycle checkpoints, and enhanced induction of apoptosis by cytarabine or DNR. These results strongly support our hypothesis that suppression of the DDR by HDACIs represents a novel mechanism underlying the antileukemic activities of this class of drugs, and their cooperativity with DNA damaging agents in AML cells.

## Supporting Information

Figure S1 **Representative Annexin V/PI dot plots and PI histograms.** THP-1 cells were treated with cytarabine or DNR, alone in combination with panobinostat (pan) for 48 h. Apoptosis events were determined by annexin V/PI staining and flow cytometry anlaysis. Representative dot plots from one experiment are shown; no drug control (**panel A**), 10 nM panobinostat (**panel B**), 4 µM cytarabine (**panel C**), 25 nM DNR (**panel D**), cytarabine plus panobinostat (**panel E**), and DNR plus panobinostat (**panel F**). The remaining cells were fixed in ethanol and cell cycle distribution was determined by PI staining and flow cytrometry analysis. Representative histograms from one experiment are shown; no drug control (**panel G**), 10 nM panobinostat (**panel H**), 4 µM cytarabine (**panel I**), 25 nM DNR (**panel J**), cytarabine plus panobinostat (**panel K**), and DNR plus panobinostat (**panel L**).
(PPTX)

Figure S2 **Panobinostat cooperates with cytarabine or DNR in inducing DNA DSBs and apoptosis, and abrogates S and/or G2/M cell cycle checkpoint activation induced by cytarabine or DNR in U937 and CTS AML cells.** U937 and CTS cells were treated with cytarabine or DNR, alone or in combination with panobinostat (10 nM) for 48 h. Early and late apoptosis events were determined by annexin V/PI staining and flow cytometry analysis (**Panels A&B**). Whole cell lysates were subjected to Western blotting (**Panels C&D**). Cell cycle distribution was determined by PI staining and flow cytometry analysis (**Panels E&F**). ***indicates p<0.0005.
(PPTX)

Figure S3 **Cell cycle distribution following cytarabine or daunorubicin treatment in THP-1 BRCA1-, CHK1-, and RAD51-shRNA knockdown cells.** THP-1 cells were infected with BRCA1-, CHK1-, RAD51-, or NTC-shRNA lentivirus overnight. The cells were washed three times with complete medium and cultured in virus-free complete medium for up to 72 h. The cells were then treated with 25 nM DNR or 4 µM ara-C for 48 h. Cell cycle distribution was determined by PI staining and flow cytometry analysis.
(PPTX)

Figure S4 **Overexpression of CHK1 causes resistance to panobinostat and significantly attenuates apoptosis induced by the combination of panobinostat and DNR or cytarabine (to a lesser extent) THP-1 cells.** THP-1 cells were infected overnight with CHK1 or RFP cDNA expression lentivirus. The cells were selected with blasticidin to generate stable clones of RFP (designated THP-1/RFP cells) or CHK1 (designated THP-1/CHK1 cells). Whole cell lysates of THP-1/RFP or THP-1/CHK1 were subjected to Western blotting **(Panel A**). THP-1/RFP or THP-1/CHK1 cells were treated with cytarabine or DNR alone or in combination with panobinostat for 48 h. Early and late apoptosis events were determined by annexin V/PI staining and flow cytometry analysis (**Panel B**). Whole cell lysates were subjected to Western blotting to measure γH2AX, CHK1, or β-actin (**Panels C&D**).
(PPTX)

Table S1 **Patient Characteristics.**
(DOC)

Table S2 **Summary of primers used for real-time RT-PCR for E2F1 ChIP.**
(DOC)

Table S3 **Mean survival of NSG mice bearing AML xenografts treated with cytarabine and panobinostat alone or in combination.**
(DOC)
