# Supplementary material for: Panobinostat Enhances Cytarabine and Daunorubicin Sensitivities in AML Cells through Suppressing the Expression of BRCA1, CHK1, and Rad51
Source: PLoS One. 2013 Nov 11;8(11):e79106. doi: 10.1371/journal.pone.0079106 (PMC3823972; doi:10.1371/journal.pone.0079106)
Supplement: Table S1 — Patient Characteristics. (DOC) [file pone.0079106.s005.doc]

**Table S1. Patient Characteristics**

| **Patient** | **Age** |  |  |  |
| --- | --- | --- | --- | --- |
| **Sample** | **(years)** | **Sex** | **Race** | **FAB** |
| A30074 | 15 | M | Caucasian | M5 |
| A30310 | 9 | M | Caucasian | M2 |
| A30320 | 16 | F | NA | NA |
| A30321 | 15 | M | Middle Eastern | M5 |
| A30322 | NA | F | Black/African American | M4 |
| A30323 | 0.7 | M | Caucasian | M4/M5 |
| A30326 | 2 | M | Caucasian | M3 |
| A30329 | NA | NA | NA | NA |
| A30338 | 8 | F | Caucasian | M4/M5 |

NA – not available
