# Supplementary material for: Panobinostat Enhances Cytarabine and Daunorubicin Sensitivities in AML Cells through Suppressing the Expression of BRCA1, CHK1, and Rad51
Source: PLoS One. 2013 Nov 11;8(11):e79106. doi: 10.1371/journal.pone.0079106 (PMC3823972; doi:10.1371/journal.pone.0079106)
Supplement: Table S2 — Summary of primers used for real-time RT-PCR for E2F1 ChIP. (DOC) [file pone.0079106.s006.doc]

**Table S2. Summary of primers used for real-time RT-PCR for E2F1 ChIP**

| Gene Promoter | Forward | Reverse | Size, bp |
| --- | --- | --- | --- |
| *BRCA1* | 5' CTTAGCGGTAGCCCCTTGGTTTCC 3' | 5' CGCCAGTACCCCAGAGCATCAC 3' | 238 |
| *RAD51* | 5' TTGAATTAGTCCTTACGCAAAAAG 3' | 5' CCTTAGGGCTCGGTCTCTG 3' | 233 |
| *CHK1* | 5' ATTTGCGTTGTAAGATTTATTTTG 3' | 5' GCGAGCAGTTTATGTTGGA 3' | 199 |
| unrelated region | 5' TTGCCTTATTTCCAAGTTCTCAGT 3' | 5' CGCCTTTCCAGTTTCTCAGA 3' | 239 |
