# Supplementary material for: Panobinostat Enhances Cytarabine and Daunorubicin Sensitivities in AML Cells through Suppressing the Expression of BRCA1, CHK1, and Rad51
Source: PLoS One. 2013 Nov 11;8(11):e79106. doi: 10.1371/journal.pone.0079106 (PMC3823972; doi:10.1371/journal.pone.0079106)
Supplement: Table S3 — Mean survival of NSG mice bearing AML xenografts treated with cytarabine and panobinostat alone or in combination. (DOC) [file pone.0079106.s007.doc]

**Table S3. Mean survival of NSG mice bearing AML xenografts treated with cytarabine and** panobinostat alone or in combination

| Treatment Group | Drug Schedule | # of mice | # of Survival at day 22 | # of Survival at day 40 | Mean Survival (day) |
| --- | --- | --- | --- | --- | --- |
| Vehicle | Daily x 5 x 3 weeks | 10 | 0 | 0 | 20 |
| 5mg/kg Panobinostat | Daily x 5 x 3 weeks | 8 | 6 | 0 | 22 |
| 6.25mg/kg Ara-C | Daily x 5 x 4 weeks | 10 | 10 | 1 | 37 |
| Combination | 5mg/kg Pano Daily x 5 x 3 weeks | 8 | 8 | 7 | 44 |
|  | 6.25mg/kg Ara-C Daily x 5 x 4 weeks |  |  |  |  |
